# Decolonising informed consent in global mental health research

**DOI:** 10.1371/journal.pmen.0000535

**Published:** 2026-01-23

**Authors:** Sandy ElChaar, Emily Oliver, Felicity L. Brown, Bayard Roberts

**Affiliations:** 1 Research and Development Department, War Child Alliance, London, United Kingdom; 2 BeyondText, London, United Kingdom; 3 UNICEF, Paris, France; 4 Faculty of Public Health and Policy, London School of Hygiene and Tropical Medicine, London, United Kingdom; PLOS: Public Library of Science, UNITED KINGDOM OF GREAT BRITAIN AND NORTHERN IRELAND

## Introduction

Despite decades of critique, many global mental health studies continue to reproduce colonial dynamics, particularly through their approach to informed consent. Too often, consent is treated as a bureaucratic formality—something to be signed once and filed away—rather than a dialogical and ongoing process [1]. This mirrors broader patterns of exploitation in research, where vulnerable participants provide labour, data, or lived experience but receive little in return [2]. Scholars have argued that such practices reflect a failure of justice and reciprocity: researchers “take without giving back,” leaving participants with few benefits [3].

These dynamics are especially visible in global mental health, where colonial logics often underpin knowledge production, privileging external researchers’ agendas over local priorities. As Norval and Henderson [4] show, even in digital contexts, informed consent is frequently compromised, with conditions of voluntariness and understanding unmet. If we are serious about decolonizing mental health research, we must move beyond tokenistic consent. Instead, researchers should commit to continuous, culturally relevant consent—a dialogical process that recognizes participants’ agency, adapts to evolving circumstances, and ensures meaningful benefit-sharing. This shift is not merely an ethical imperative; it is also a methodological necessity for producing trustworthy and, contextually grounded research.

Traditional consent models are often developed before researchers even set foot in the communities they seek to study. Consent forms are drafted in distant institutional offices, shaped by Western assumptions about literacy, individual autonomy, and legalistic procedure [5]. When introduced in the field, these documents rarely reflect local cultural contexts, languages, or collective decision-making processes [1].

Such practices perpetuate extraction. Researchers accrue publications, career advancement, and funding, while participants are left with little agency and no assurance that their contributions will benefit them or their communities [2]. Evidence from humanitarian healthcare illustrates this imbalance: a systematic review found that while conflict-affected and forcibly displaced populations were frequently engaged to deliver interventions, they were rarely involved in framing problems or designing solutions [6]. Engaging communities only at the delivery stage is tokenistic, serving to legitimize external agendas rather than redistributing power.

Continuous consent can be defined as “an ongoing, dialogical process in which individuals from diverse cultural backgrounds are provided with information in a manner that is culturally sensitive, clear, and accessible” [1]. Unlike static consent, which is obtained once and rarely revisited, continuous consent unfolds across the entire research lifecycle—beginning at the proposal stage, reaffirmed during recruitment, revisited in the midst of data collection, and sustained through the dissemination of findings. This process requires researchers to check in regularly with participants, ensuring that they still wish to take part and that the process remains respectful and meaningful. By shifting from one-off agreements to iterative engagement, continuous consent not only protects autonomy but also builds trust, enhances comprehension, and prevents communities from being locked into decisions made under unequal conditions.

A recent study in Lebanon sought to move beyond abstract principles and demonstrate what continuous, culturally relevant consent can look like in practice. Working with Palestinian, Syrian, and Lebanese participants in the Beqaa and North Lebanon regions, researchers combined Design Thinking and Participatory Action Research to co-develop context-specific consent guidelines [1]. This collaborative process generated a set of practical tools. Participants emphasized the need for simple and accessible language, the use of audio-visual formats, clarity about time commitments, and opportunities to withdraw without stigma. Importantly, these solutions were not externally imposed but co-designed, embodying the spirit of decolonial consent by centering participant agency and cultural realities.

Other models reinforce this. In digital health, dynamic consent allows participants to update their choices over time [1]. Humanitarian initiatives such as the HESHIMA Project and the Service User Participation Toolkit for Mental Health Services, emphasise accessibility, inclusion, and the need to avoid tokenism [7]. To sustain these practices, institutions must embed decolonial consent training into research curricula and professional development. Without structural reinforcement, good intentions risk dissipating.

Continuous, culturally relevant consent is more than a methodological innovation. It is a moral and political necessity for decolonizing mental health research. It respects participants not as passive data points but as active partners, knowledge holders, and co-authors of change.

Consent in research is not merely a procedural safeguard; it also carries epistemic significance, shaping who is recognized as a legitimate producer of knowledge and who is relegated to the status of a “data source.” However, it is acknowledged that improving consent procedures alone cannot address these epistemological hierarchies. Decolonizing consent is therefore only one step - albeit an important one - toward reconfiguring research relationships and redistributing authority within global mental health research [8,9].

Beyond consent, broader epistemic and participatory challenges also need to be addressed. Mad Studies, grounded in the testimonies and critiques of psychiatric survivors, provides an important corrective to dominant paradigms. It highlights how global mental health initiatives often impose diagnostic categories and treatment models developed in the Global North, while marginalizing experiential knowledge and local epistemologies [10]. Parallel debates in the field of epistemic justice emphasize that communities should not only participate in data provision but also be acknowledged as knowers and co-authors, with the power to shape research questions, methods, and interpretations [11].

Within this framing, continuous consent operates as a mechanism of epistemic decolonization. By granting participants ongoing control over how their narratives are collected, interpreted, and disseminated, it resists the historical pattern of researchers unilaterally determining how community experiences are framed and used. In this sense, continuous consent functions not only as an ethical safeguard but also as a necessary tool for redistributing epistemic authority in decolonized research practices.

### Practical mechanisms to implement continuous consent

For researchers committed to decolonial practice, continuous consent can be operationalized through a clear, replicable framework:

- Project planning: Engage communities before finalising research questions so that their voices shape the framing [1,6].
- Co-design workshops: Develop consent materials collaboratively, using visual, oral, and interactive methods where appropriate [1].
- Staged consent: Revisit consent iteratively—at recruitment, during data collection, and at dissemination—to ensure ongoing relevance [3].
- Teach-back method: Ask participants to restate in their own words what they understand, checking for clarity [12].
- Feedback loops: Establish accessible channels for participants to raise concerns and revise their involvement [8].
- Data return: Share findings in culturally meaningful formats—stories, community meetings, infographics—rather than only academic outputs [11].

The path forward necessitates that:

- **Funders** must require budgets for ongoing consent processes.
  - **Institutions** must embed decolonial consent into training and ethical review.
  - **Journals** must spotlight methodological innovations that challenge extractive norms and expect that studies embed decolonial consent within their procedures.

Only once the above is in place can research consent move from extractive encounters to genuine partnerships, building knowledge that both serves—and is shaped by—the communities it seeks to help.
